# Factors influencing hypothermia in very low/extremely low birth weight infants: a meta-analysis

**DOI:** 10.7717/peerj.14907

**Published:** 2023-02-20

**Authors:** Qinchuan Shi, Jingjing Zhang, Chong Fan, Aixia Zhang, Zhu Zhu, Yingying Tian

**Affiliations:** 1 Pediatric Surgery, Women’s Hospital of Nanjing Medical University, Nanjing Maternity and Child Health Care Hospital, Nanjing, Jiangsu, PR China; 2 Obstetrics, Women’s Hospital of Nanjing Medical University, Nanjing Maternity and Child Health Care Hospital, Nanjing, Jiangsu, PR China; 3 Emergency Medicine, Women’s Hospital of Nanjing Medical University, Nanjing Maternity and Child Health Care Hospital, Nanjing, Jiangsu, PR China; 4 Nursing, Women’s Hospital of Nanjing Medical University, Nanjing Maternity and Child Health Care Hospital, Nanjing, Jiangsu, PR China; 5 Special Section, Women’s Hospital of Nanjing Medical University, Nanjing Maternity and Child Health Care Hospital, Nanjing, Jiangsu, PR China

**Keywords:** Very low birth weight infants, Extremely low birth weight infants, Hypothermia, Meta-analysis

## Abstract

**Introduction:**

Previous studies have explored factors that influence the occurrence of hypothermia in very low/extremely low birth weight (VLBW/ELBW) infants, but the factors associated with hypothermia in VLBW or ELBW infants remain inadequately evaluated due to limited prospective data and inconsistency in study populations. Therefore, it is necessary to systematically evaluate the risk factors of hypothermia in VLBW/ELBW infants in order to provide a theoretical basis for clinical practice.

**Methods:**

PubMed and other databases were used to search for case-control or cohort studies on factors influencing the occurrence of hypothermia in VLBW/ELBW infants. The search time was set from database creation to June 30th, 2022. Literature screening, quality evaluation, and data extraction were performed independently by two investigators according to predefined inclusion and exclusion criteria. Meta-analysis was performed using RevMan 5.3.

**Results:**

A total of 10 papers were finally included in this study and 12 factors were established by meta-analysis: body weight (six papers), failure to keep warm in time (three papers), neonatal resuscitation (seven papers), gestational age (three papers), premature rupture of membranes (three papers), maternal combined complications (four papers), cesarean section (six papers), antenatal steroids (four papers), multiple birth (two papers), small for gestational age (two papers), 1 min Apgar score (three papers), and 5 min Apgar score (three papers). Since only one study included race, age (hour), socio-economic status, and spontaneous labor, these factors could not be fitted into RevMan 5.3 for the analysis.

**Conclusion:**

Although there were differences in the study design of the included literature, the influencing factors described in each study were relatively similar. The influencing factors identified in this study may contribute to the construction of related intervention strategies for hypothermia in VLBW/ELBW infants.

## Introduction

Neonatal hypothermia is characterized by an abnormal thermal state in which a newborn’s body temperature falls below 36.5 °C (97.7 °F). Its prevalence ranges from 31% to 78% worldwide (Mank et al., 2016; Caldas et al., 2018). Based on core body temperature, the World Health Organization (WHO) categorizes hypothermia into three groups: cold stress (36.0 °C to 36.4 °C, 96.8 °F to 97.5 °F), moderate hypothermia (32.0 °C to 35.9 °C, 89.6 °F to 96.6 °F), and severe hypothermia (<32.0 °C, <89.6 °F) (Feldman et al., 2016). Very low/extremely low birth weight (VLBW/ELBW) infants have a severely limited ability to maintain core body temperature compared to healthy newborns, and so have limited vasoconstriction capacity, higher surface to mass ratio, and less brown fat deposition, which is essential for non-fermentative thermogenesis (Fairchild et al., 2011; Fawcett, 2014). Hypothermia occurs soon following birth in preterm infants, especially in VLBW and ELBW infants, and remains a worldwide problem that can lead to a variety of adverse consequences (Nemeth, Miller & Bräuer, 2021). It has been reported that every 1 °C drop in neonatal body temperature could increase the risk of death by 28% (OR = 1.28, 95% CI [1.16~1.42]) (Laptook et al., 2018) and the occurrence of neonatal hypothermia is significantly associated with the incidence of necrotizing entero-colitis (NEC), intraventricular hemorrhage (IVH), and late-onset sepsis (LOS) (Jakuskiene, Daugeliene & Vollmer, 2009; Shah et al., 2014; Cavallin et al., 2020). Therefore, it is urgent to provide theoretical guidance to improve the survival rate, quality of survival, and administrated direction for VLBW/ELBW infants. Although several works (Almeida et al., 2009; Ali, Osman & Mustafa, 2022; Wilar & Lestari, 2022) have focused on influencing factors, the factors associated with hypothermia of VLBW and ELBW infants remain under-evaluated due to limited prospective data and the inconsistency of the study population. Therefore, there is still a lack of clinical consensus on the risk factors associated with hypothermia due to few existing published studies and recommendations to support healthcare professionals’ decision-making. In this study, we conducted a systematic search for studies on factors influencing the occurrence of hypothermia in VLBW and ELBW infants. Additionally, we performed a meta-analysis of the relevant factors in order to obtain scientifically valid results to guide clinical practice.

## Materials and Methods

### Literature search and screen

The relevant literature were searched using a combination of subject terms and free words *via* the databases Embase, Cochrane Library, PubMed, and Web of Science. The search terms were: very low birth weight infant(s), very low birth weight, extremely low birth weight infant(s), hypothermia(s), accidental hypothermia(s), risk, relative risk, cohort studies, cohort. The search time was set from the establishment of the database to June 30, 2022. Inclusion criteria were followed using a clear definition of the concept of hypothermia, VLBW/ELBW infants, and relevant risk factors. Exclusion criteria consisted of normal weight neonates, unavailability of full text, unavailability of Odds Ratio (OR)/Risk Ratio (RR) values for relevant factors, and type of literature (emails, conference articles, reviews, systematic reviews, or meta-analyses). The factors and search terms are shown in Table 1 and detailed search strategies are shown in Appendix 1.

**Table 1:** Factors and search terms.

Factors	Search terms
Very low weight infants	((((“Infant, Very Low Birth Weight”[Mesh]) OR ((((((Very-Low-Birth-Weight Infant[Title/Abstract]) OR (Infant, Very-Low-Birth-Weight[Title/Abstract])) OR (Infants, Very-Low-Birth-Weight[Title/Abstract])) OR (Very Low Birth Weight Infant[Title/Abstract])) OR (Very-Low-Birth-WeightInfants[Title/Abstract])) OR (Very Low Birth Weight[Title/Abstract])))
Extremely low birth weight infant	((“Infant, ExtremelyLow Birth Weight”[Mesh]) OR (Extremely Low Birth Weight Infant[Title/Abstract])))
Hypothermia	((“Hypothermia”[Mesh]) OR (((((Hypothermias[Title/Abstract]) OR (Hypothermia, Accidental[Title/Abstract])) OR (Accidental Hypothermia[Title/Abstract])) OR (Accidental Hypothermias[Title/Abstract])) OR (Hypothermias, Accidental[Title/Abstract]))))
Risk	((relative[Title/Abstract] AND risk*[Title/Abstract]) OR (relative risk[Text Word]) OR risks[Text Word]
Cohort	cohort studies[MeSH:noexp] OR (cohort[Title/Abstract] AND stud*[Title/Abstract]))

### Date extraction

The literature screening and data extraction were carried out individually by two researchers. Endnote software was used to eliminate duplicate literature, systematic evaluation, review, meta-analysis, and animal experiments. The remaining literature’s titles, abstracts, and full text were read through to complete the secondary screening and ambiguous literature were adjudicated by another researcher. The basic information, study characteristics, and outcome indicators of the literature were extracted by developing a literature extraction form based on the JBI evaluation manual (Peters et al., 2020).

### Quality evaluation and risk bias assessment

Since the types of literature included in this study were case-control or cohort studies, the Newcastle-Ottawa Scale (NOS) was used to evaluate the quality (Stang, 2010). This scale assesses the quality level of case-control studies or cohort studies in terms of study population selection, comparability, and exposure (outcome), with a total maximum score of nine. Original literature with a score greater than or equal to six was included in the subsequent meta-analysis. Detailed information on quality assessment and risk bias assessment are provided in Appendix 2.

### Analysis

Data analysis was executed using Review Manager 5.3. The RR or OR values were applied as the effect analysis statistic. We calculated a 95% confidence index (CI) for each effect size based on the dichotomous variables of the current study outcome. Moreover, heterogeneity of the enrolled studies was evaluated using a Chi-square test and *I*^2^ values. If *P* > 0.1 and *I*^2^ < 50%, it indicated that the heterogeneity among studies was small and it was appropriate to combine effect size using a fixed-effect model; if *P* < 0.1 and *I*^2^ > 50%, it indicated that the heterogeneity among studies was large and it was appropriate to combine effect size using a random-effect model. Begg’s test was used to test the publication bias of the included studies and *P* < 0.05 was considered statistically significant.

## Results

**Figure 1:**
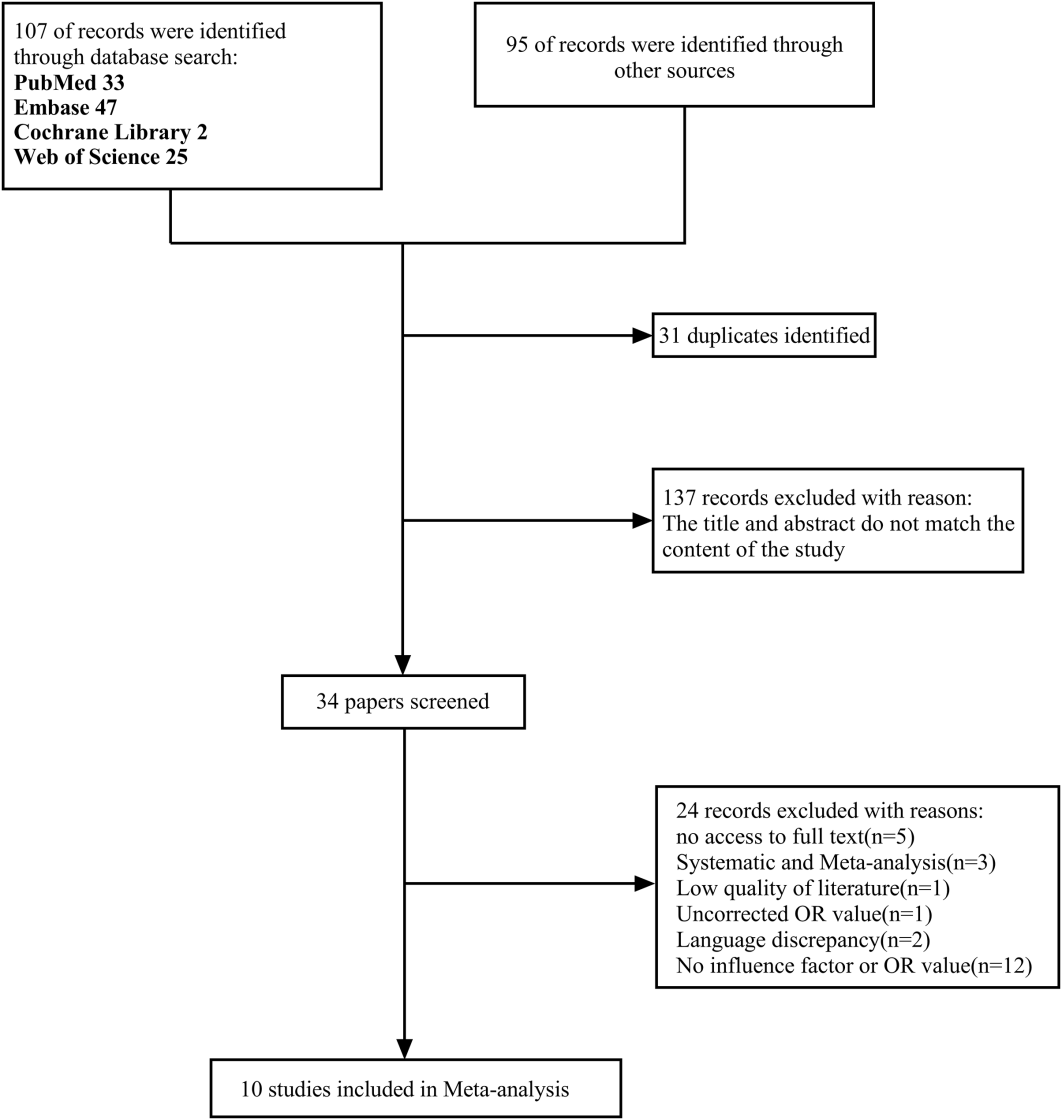
Screening process. A total of 202 papers were initially obtained, and 10 papers were finally included after double screening.

Initially, 202 relevant papers were retrieved, 31 duplicates were excluded by Endnote software, and 137 papers were excluded after reading the titles and abstracts and according to the inclusion and exclusion criteria set for this study. A secondary screening of the remaining 34 papers was performed, and 10 papers were finally included. Reasons for exclusion included unavailability of full text (*n* = 5), NOS score <6 (*n* = 1), systematic evaluation and meta-analysis (*n* = 3), unadjusted OR values (*n* = 1), language discrepancy (*n* = 2), and no clearly defined influence factors or OR values (*n* = 12). The literature screening process is shown in Fig. 1. Because the *P* value of Begg’s test was greater than 0.05, this demonstrated that the literature included in this study had no publication bias. This study has been registered on the PROSPERO platform under registration number CRD42022342782.

### Study characteristics

The basic characteristics of the involved literature are shown in Table 2. Among these 10 studies, five were from China, and the other five studies were from the US, Brazil, Bangladesh, Sweden, and South Korea.

**Table 2:** Summary of influencing factors.

Inclusion of literature	Country	Type of study	Sample capacity		Influencing factors	NOS score
			Cases	Controls		
Gao et al. (2022)	China	Case control study	125	122	①②③	7
Li et al. (2013)	China	Case control study	181	106	④⑤⑥⑦	7
Zhou et al. (2010)	China	Case control study	190	134	①③⑯⑰	7
Dong (2021)	China	Case control study	60	40	③④⑤⑥	7
Yu et al. (2020)	China	Cohort study	1,100	147	①③④⑤⑥⑧⑨⑩⑫	9
de Almeida et al. (2014)	Brazil	Cohort study	593	754	⑦⑨⑩	9
Akter, Parvin & Yasmeen (2013)	Bangladesh	Cohort study	785	1,525	①③⑤⑬⑭	9
Miller, Lee & Gould (2011)	USA	Cohort study	4,932	3,850	①②③④⑤⑥⑦⑨⑪⑮	9
Wilson et al. (2018)	Sweden	Cohort study	3,132	2,729	⑦	9
Lee et al. (2019)	Korea	Cohort study	3,462	1,211	①③⑤⑥⑧⑨⑩⑫	9

**Note:**

① Birth weight; ② Multiple births; ③ Neonatal resuscitation; ④ Antenatal steroids; ⑤ Cesarean section; ⑥ Apgar score; ⑦ Failure to keep warm in time; ⑧ Small for gestational age (SGA); ⑨ Maternal combined complications; ⑩ Gestational age; ⑪ Race; ⑫ Premature rupture of membranes (PROM); ⑬ Newborn age (hour); ⑭ Socioeconomic status; ⑮ Spontaneous delivery; ⑯ Not born in our hospital; ⑰ Born in the cold season.

### Meta-analysis results

After data extraction and meta-analysis, a total of 12 factors were summarized in this study: 1 min Apgar score, 5 min Apgar score, small for gestational age (SGA), maternal combined complications, birth weight, multiple births, Cesarean section, premature rupture of membranes (PROM), failure to keep warm in time, neonatal resuscitation, antenatal steroids, and gestational age. As shown in Table 3, we found statistical differences in failure to keep warm in time, neonatal resuscitation, gestational age, maternal combined complications, Cesarean section, and 5 min Apgar score.

**Table 3:** Meta-analysis results.

Influencing factors	Study (*n*)	*I*^2^ (%)	Effect model	OR (95% CI)	*P*
Body weight	6	92	Random	1 [1–1]	0.32
Failure to keep warm in time	3	86	Random	1.97 [1.19–3.28]	0.008
Neonatal resuscitation	7	75	Random	1.86 [1.43–2.43]	<0.001
Gestational age	3	27	Fixed	1.1 [1.09–1.1]	<0.001
Premature rupture of membranes	3	0	Fixed	1.08 [0.93–1.25]	0.32
Maternal combined complications	4	85	Random	1.28 [1.01–1.62]	0.04
Cesarean section	6	89	Random	1.67 [1.15–2.41]	0.006
Antenatal steroids	4	88	Random	0.78 [0.42–1.44]	0.42
Multiple birth	2	84	Random	1.33 [0.54–3.28]	0.53
Small for gestational age	2	54	Random	1.49 [0.93–2.38]	0.09
1 min Apgar score	3	91	Random	1.43 [0.96–2.14]	0.28
5 min Apgar score	3	65		1.47 [1.21–1.78]	<0.001

### Body weight

**Figure 2:**
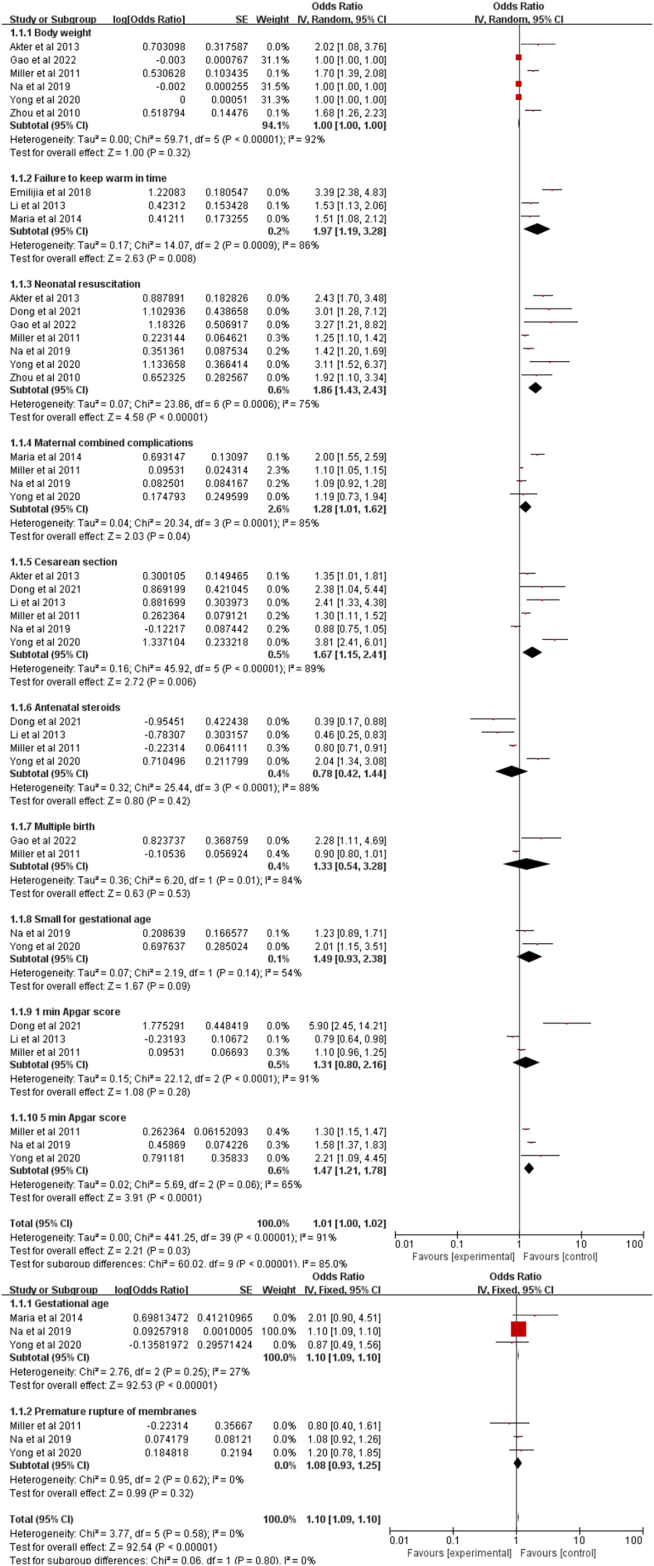
Forest plot of all influencing factors. A total of 12 influencing factors were included in the meta-analysis, and the results showed a statistical difference in failure to keep warm in time, neonatal resuscitation, gestational age, maternal combined complications, Cesarean section, and 5 min Apgar score.

Of the included papers, six involved the effect of body weight on the occurrence of hypothermia (Zhou et al., 2010; Miller, Lee & Gould, 2011; Akter, Parvin & Yasmeen, 2013; Lee et al., 2019; Yu et al., 2020; Gao et al., 2022), and the overall effect (*P* = 0.32, 95% CI [1.00–1.00]) of body weight on the occurrence of hypothermia was determined to be not statistically different by meta-analysis (Fig. 2).

### Failure to keep warm in time

The forest plot for the failure to keep warm in time has also been reported in previous research and three studies (Miller, Lee & Gould, 2011; Li et al., 2013; Wilson et al., 2018) were included in the final analysis (Fig. 2). The forest plot shows that failure to keep warm in time is one of the influencing factors for the occurrence of hypothermia.

### Neonatal resuscitation

The results of the meta-analysis of neonatal resuscitation are shown in Fig. 2. In this part, seven studies (Zhou et al., 2010; Miller, Lee & Gould, 2011; Akter, Parvin & Yasmeen, 2013; Lee et al., 2019; Yu et al., 2020; Dong, 2021; Gao et al., 2022) were contained for the final analysis. The OR value for neonatal resuscitation was 1.86 (*P* < 0.0001, 95% CI [1.43–2.43]).

### Gestational age

Three papers (Miller, Lee & Gould, 2011; Lee et al., 2019; Yu et al., 2020) were ultimately selected for the analysis of gestational week on hypothermia (Fig. 2). A fixed effects model was used for the investigation because of high heterogeneity (*I*^2^ = 27%). As a consequence, the calculated OR value for gestational age was 1.10 (*P* < 0.0001, 95% CI [1.09–1.10]).

### Premature rupture of membranes

Premature rupture of membranes, a common risk factor for adverse outcomes in both obstetrics and pediatrics, has been shown to be related to the occurrence of hypothermia. Three papers (Miller, Lee & Gould, 2011; Lee et al., 2019; Yu et al., 2020) discussed the effect of premature rupture of membranes on the occurrence of hypothermia (Fig. 2). The results implied no significant difference for premature rupture of membranes on the occurrence of hypothermia (*P* = 0.32, 95% CI [0.93–1.25]).

### Maternal combined complications

Four papers (Miller, Lee & Gould, 2011; de Almeida et al., 2014; Lee et al., 2019; Yu et al., 2020) studied the effect of maternal combined complications on the occurrence of hypothermia. The results indicated that the OR value for maternal combined complications was 1.28 (*P* = 0.04, 95% CI [1.01–1.62]). The integration results are shown in Fig. 2.

### Cesarean section

Six studies (Miller, Lee & Gould, 2011; Akter, Parvin & Yasmeen, 2013; Li et al., 2013; Lee et al., 2019; Yu et al., 2020; Dong, 2021) on Cesarean section were used in our analysis (Fig. 2). The combined results of the random effects model showed that the OR value for Cesarean section was 1.67 (*P* = 0.006, 95% CI [1.15–2.41]).

### Antenatal steroids

The use of antenatal steroids is common, controversial, and has been connected with the occurrence of hypothermia. In this section, four studies (Miller, Lee & Gould, 2011; Li et al., 2013; Yu et al., 2020; Dong, 2021) were recruited for final analysis (Fig. 2). The results showed no significant effect of antenatal steroids on the occurrence of hypothermia after random effects model integration.

### Multiple births

Of the 10 included papers, two studies (Miller, Lee & Gould, 2011; Gao et al., 2022) were on the effects of multiple births on hypothermia in VLBW/ELBW infants (Fig. 2). As a result, the OR value for multiple births was 1.33 (*P* = 0.53, 95% CI [0.54–3.28]).

### Small for gestational age (SGA)

Being SGA is associated with many adverse outcomes, including hypothermia. In this article, two studies (Lee et al., 2019; Yu et al., 2020) were included in the meta-analysis (Fig. 2). The forest plot manifested that there was no significant difference for SGA on the occurrence of hypothermia (*P* = 0.09, 95% CI [0.93–2.38]).

### Apgar score

Apgar score is considered a vital indicator for the evaluation of newborns. Five studies (Miller, Lee & Gould, 2011; Li et al., 2013; Lee et al., 2019; Yu et al., 2020; Dong, 2021) were included in the ultimate analysis (Fig. 2). The results showed that a 1 min Apgar score was not statistically significant for the occurrence of hypothermia (*P* = 0.28, 95% CI [0.8–2.16]), but a 5 min Apgar score was an influencing factor for the occurrence of hypothermia (*P* < 0.001, 95% CI [1.21–1.78]).

### Other factors

Four other relevant factors could not be meta-analyzed because were included in only one study: race 1.3 (1.1~1.5), age 2.25 (1.13~4.47), socio-economic status 2.76 (1.56~4.9), and spontaneous labor 0.8 (0.7~0.9).

## Discussion

Failure to keep warm in time involves low room temperature, prolonged time in the room, and no systematic use of hypothermia prevention. Studies have confirmed that for every 2–3 °C drop in deep body temperature, skin temperature drops by 4–6 °C within 30 min of birth. Therefore, to prevent rapid evaporation of heat from newborns, WHO recommends that room temperature should be maintained at 25 °C (Russo et al., 2014). Prolonged time in the room is a risk factor for hypothermia in VLBW/ELBW infants, and may lead to heat evaporation during routine nursing operations and resuscitation due to varying levels of proficiency of health care workers and failure to keep VLBW/ELBW infants warm in a timely manner (Reilly et al., 2015). Hypothermia prevention strategies mainly include the use of polyethylene or polyurethane plastic bags (caps) with variable heat mattresses, and studies have shown that the implementation of these measures can reduce evaporation and transepidermal water loss and maintain constant body temperature by maintaining skin moisture in VLBW/ELBW infants (Sharma et al., 2020).

Neonatal resuscitation includes resuscitation needed, intubation at delivery room, and history of neonatal asphyxia. Neonatal resuscitation may cause hypothermia due to the lack of cerebral blood flow, causing damage of brain tissue and the thermoregulatory center. On the other hand, the main focus of the medical staff during the resuscitation of an infant with asphyxia is on saving the infant’s life, but this may result in prolonged exposure and increased heat evaporation (Zayeri et al., 2007; Ogunlesi et al., 2008).

It has been confirmed that VLBW/ELBW infants have a significant difference in body weight compared to full term infants due to their younger gestational age (Pinheiro et al., 2014). Body weight is closely related to heat production, storage, and thermoregulation in VLBW/ELBW infants. In terms of heat production, these infants have lower birth weight and less brown fat, which is the main source of heat production in infants, and therefore, they do not produce enough heat compared to normal weight infants. The immature development of organs leads to the imperfect development of the thermoregulation center, which aggravates the occurrence of hypothermia (Boo & Guat-Sim Cheah, 2013; Tay et al., 2019).

In the literature we examined, maternal combined complications included gestational hypertension, gestational diabetes mellitus, and chorioamnionitis. Maternal combined complications are often associated with a risk of preterm delivery and related medications, such as hormones and magnesium sulfate, that are used more frequently and can easily lead to muscle relaxation and respiratory depression, increasing the risk of resuscitation and exposure of the child (Greenberg et al., 2011).

The results of this meta-analysis indicated that Cesarean delivery is one of the risk factors for the development of hypothermia in VLBW/ELBW infants, which may be caused by the fact that maternal heat can be generated during the second stage of labor during vaginal delivery by exertion, which promotes the increase of neonatal body temperature. At the same time, the use of anesthetic drugs during Cesarean delivery is not conducive to the recovery of maternal and neonatal thermoregulatory center functions after surgery (Johannsen, Vochem & Neuberger, 2017).

Our meta-analysis suggested that the 5 min Apgar score was a factor influencing the occurrence of hypothermia in VLBW/ELBW infants. Hypothermia may be a result of the neonatal thermoregulatory center being impaired due to asphyxia or other complications. It is also possible that hypothermia is caused by neglecting the application of warming measures during resuscitation (Croop et al., 2020).

Although this study was based on an evidence-based correlation approach to analyze the factors influencing hypothermia in VLBW/ELBW infants, there were several limitations in this meta-analysis. Above all, there was a large heterogeneity across some of the factors. Though we attempted to explore the sources of heterogeneity using subgroup analysis, the effect was minimal. After research and discussion, the reasons may be as follows: first, there were differences in the diagnosis of hypothermia among studies. Although WHO proposed a threshold of 36.5 °C for hypothermia, some of the studies used 36.0 °C or 35 °C as the threshold, which increased the heterogeneity among studies due to different inclusion criteria. In addition, there was inconsistency in the way neonatal temperature was measured. Among the included studies, neonatal temperature measurement methods included rectal, axillary and/or skin, and the inconsistency of temperature measurement methods may have led to differences in temperature reporting between studies, thus increasing the heterogeneity of the results of this meta-analysis. On the whole, there were large differences in temperature variation among different ethnic groups. The American Collaboration for Advancing Pediatric Quality Measures (CAPQuaM) confirmed by analyzing data on newborns in New York State that there were differences in temperature variation among different ethnic groups of newborns, and black infants were more likely to have hypothermia compared with white infants (Lawrence, 2016). In the literature included in this study, the studies covered Asian, European, and American populations, and the differences in body mass of different ethnic groups may have affected the OR values of influencing factors in risk prediction models, thus leading to greater heterogeneity. Second, although the overall risk bias of this study was assessed as low by NOS, the different diagnostic criteria for hypothermia among studies made it possible that there was some information bias in this study. At the same time, because the influencing factor meta-analysis methodology only extracted the OR values for analysis, this study team was unable to explore the potential interactions among the influence factors, indicating that the final results of the influence factor analysis may have some confounding bias. Third, the number of included studies was limited, which may lead to imprecise results of the meta-analysis. The reason for this is the special population of VLBW/ELBW infants, which makes it difficult to obtain more clinical cohort data for research and analysis. Furthermore, due to the particularity of the population, relevant research focused on clinical nursing quality improvement projects, and the discussion and quantification of influencing factors were not involved.

## Conclusion

Through literature generalization, we determined a total of 16 factors influencing hypothermia in VLBW/ELBW infants. Of these, four influencing factors (race, age, socio-economic status, and spontaneous labor) were included in only one study, and so the meta-analysis was not performed on them. The remaining 12 factors were included in the meta-analysis, and the results showed that failure to keep warm in time, neonatal resuscitation, gestational age, maternal combined complications, Cesarean section and 5-min Apgar score were contributing factors for hypothermia in VLBW/ELBW infants.

## Supplemental Information

10.7717/peerj.14907/supp-1 Supplemental Information 1 Search strategy. The appendix contains search strategies for 4 databases: PubMed, Embase, Cochrane Library and Web of Science.

10.7717/peerj.14907/supp-2 Supplemental Information 2 Quality assessment and bias analysis. The appendix contains the results of the quality evaluation and risk bias assessment of the included literature.

10.7717/peerj.14907/supp-3 Supplemental Information 3 PRISMA checklist.

10.7717/peerj.14907/supp-4 Supplemental Information 4 Literature data extraction information and software operation procedures.

10.7717/peerj.14907/supp-5 Supplemental Information 5 Systematic review and meta-analysis rationale.

## Additional Information and Declarations

### Data Availability

The following information was supplied regarding data availability: Search strategy, quality assessment and risk bias assessment, PRISMA checklist, data, systematic review, and meta-analysis rationale are available in the Supplemental Files.

## References

[ref-1] Akter S, Parvin R, Yasmeen BHN (2013). Admission hypothermia among neonates presented to Neonatal intensive care unit. Journal of Nepal Paediatric Society.

[ref-2] Ali TAB, Osman NM, Mustafa AEM (2022). Pattern of low birth weight and early outcome of neonates admitted at neonatal unit in Omdurman Maternity Hospital from December 2019 to May 2020. Bahrain Medical Bulletin.

[ref-3] Almeida PG, Chandley J, Davis J, Harrigan RC (2009). Use of the heated gel mattress and its impact on admission temperature of very low birth-weight infants. Advances in Neonatal Care: Official Journal of the National Association of Neonatal Nurses.

[ref-4] Boo NY, Guat-Sim Cheah I (2013). Admission hypothermia among VLBW infants in Malaysian NICUs. Journal of Tropical Pediatrics.

[ref-5] Caldas JPS, Millen FC, Camargo JF, Castro PAC, Camilo A, Marba STM (2018). Effectiveness of a measure program to prevent admission hypothermia in very low-birth weight preterm infants. Jornal de Pediatria.

[ref-6] Cavallin F, Bonasia T, Yimer DA, Manenti F, Putoto G, Trevisanuto D (2020). Risk factors for mortality among neonates admitted to a special care unit in a low-resource setting. BMC Pregnancy and Childbirth.

[ref-7] Croop SEW, Thoyre SM, Aliaga S, McCaffrey MJ, Peter-Wohl S (2020). The Golden Hour: a quality improvement initiative for extremely premature infants in the neonatal intensive care unit. Journal of Perinatology: Official Journal of the California Perinatal Association.

[ref-8] de Almeida MFB, Guinsburg R, Sancho GA, Rosa IRM, Lamy ZC, Martinez FE, da Silva RPGVC, Ferrari LSL, de Souza Rugolo LMS, Abdallah VOS, de Silveira RC (2014). Hypothermia and early neonatal mortality in preterm infants. The Journal of Pediatrics.

[ref-9] Dong N (2021). Analysis on the influencing factors for occurrence of hypothermia in very low birth weight infants in neonatal intensive care unit. Clinical Medicine & Engineering.

[ref-10] Fairchild KD, Sun CC, Gross GC, Okogbule-Wonodi AC, Chasm RM, Viscardi RM (2011). NICU admission hypothermia, chorioamnionitis, and cytokines. Journal of Perinatal Medicine.

[ref-11] Fawcett K (2014). Preventing admission hypothermia in very low birth weight neonates. Neonatal Network.

[ref-12] Feldman A, De Benedictis B, Alpan G, La Gamma EF, Kase J (2016). Morbidity and mortality associated with rewarming hypothermic very low birth weight infants. Journal of Neonatal-Perinatal Medicine.

[ref-13] Gao R, Li C, Gao LL, Han MY, Xu P (2022). Study on risk factors of hypothermia at admission in very low birth weight infants and/or premature infants with gestational age less than 32 weeks. Chinese Journal of Child Health Care.

[ref-14] Greenberg MB, Penn AA, Thomas LJ, El-Sayed YY, Caughey AB, Lyell DJ (2011). Neonatal medical admission in a term and late-preterm cohort exposed to magnesium sulfate. American Journal of Obstetrics and Gynecology.

[ref-15] Jakuskiene R, Daugeliene D, Vollmer B (2009). Mortality and morbidity in low birth weight newborns in Kaunas. Developmental Medicine and Child Neurology.

[ref-16] Johannsen JKI, Vochem M, Neuberger P (2017). Does a higher ambient temperature in the delivery room prevent hypothermia in preterm infants <1500 g?. Zeitschrift fur Geburtshilfe und Neonatologie.

[ref-17] Laptook AR, Bell EF, Shankaran S, Boghossian NS, Wyckoff MH, Kandefer S, Walsh M, Saha S, Higgins R (2018). Admission temperature and associated mortality and morbidity among moderately and extremely preterm infants. Journal of Pediatrics.

[ref-18] Lawrence K (2016). Timely temperatures for all low birth weight neonates. https://www.ahrq.gov/sites/default/files/wysiwyg/pqmp/measures/acute/chipra-116-fullreport.pdf.

[ref-19] Lee NH, Nam SK, Lee J, Jun YH (2019). Clinical impact of admission hypothermia in very low birth weight infants: results from Korean Neonatal Network. Korean Journal of Pediatrics.

[ref-20] Li YM, Lin XQ, Zhang LP, Jia YS, Quan XZ, Jia XH, Lin XX, Shi HY (2013). Analysis of influencing factors of admission hypothermia in very low birth weight infants. Chinese Journal of Modern Nursing.

[ref-21] Mank A, van Zanten HA, Meyer MP, Pauws S, Lopriore E, Te Pas AB (2016). Hypothermia in preterm infants in the first hours after birth: occurrence, course and risk factors. PLOS ONE.

[ref-22] Miller SS, Lee HC, Gould JB (2011). Hypothermia in very low birth weight infants: distribution, risk factors and outcomes. Journal of Perinatology.

[ref-23] Nemeth M, Miller C, Bräuer A (2021). Perioperative hypothermia in children. International Journal of Environmental Research and Public Health.

[ref-24] Ogunlesi TA, Ogunfowora OB, Adekanmbi FA, Fetuga BM, Olanrewaju DM (2008). Point-of-admission hypothermia among high-risk Nigerian newborns. BMC Pediatrics.

[ref-25] Peters MDJ, Marnie C, Tricco AC, Pollock D, Munn Z, Alexander L, McInerney P, Godfrey CM, Khalil H (2020). Updated methodological guidance for the conduct of scoping reviews. JBI Evidence Synthesis.

[ref-26] Pinheiro JM, Furdon SA, Boynton S, Dugan R, Reu-Donlon C, Jensen S (2014). Decreasing hypothermia during delivery room stabilization of preterm neonates. Pediatrics.

[ref-27] Reilly MC, Vohra S, Rac VE, Dunn M, Ferrelli K, Kiss A, Vincer M, Wimmer J, Zayack D, Soll RF (2015). Randomized trial of occlusive wrap for heat loss prevention in preterm infants. The Journal of Pediatrics.

[ref-28] Russo A, McCready M, Torres L, Theuriere C, Venturini S, Spaight M, Hemway RJ, Handrinos S, Perlmutter D, Huynh T, Grunebaum A, Perlman J (2014). Reducing hypothermia in preterm infants following delivery. Pediatrics.

[ref-29] Shah V, Chiang J, Chung S, Ling Y (2014). To determine the incidence, risk factors and need for surgery for retinopathy of prematurity (ROP) among Very-Low-Birth-Weight (VLBW) infants weighing <1500 gms. Archives of Disease in Childhood.

[ref-30] Sharma D, Murki S, Kulkarni D, Pawale D, Vardhelli V, Anne RP, Oleti TP, Deshabhotla S (2020). The impact of a quality improvement project to reduce admission hypothermia on mortality and morbidity in very low birth weight infants. European Journal of Pediatrics.

[ref-31] Stang A (2010). Critical evaluation of the Newcastle-Ottawa scale for the assessment of the quality of nonrandomized studies in meta-analyses. European Journal of Epidemiology.

[ref-32] Tay VY, Bolisetty S, Bajuk B, Lui K, Smyth J (2019). Admission temperature and hospital outcomes in extremely preterm infants. Journal of Paediatrics and Child Health.

[ref-33] Wilar R, Lestari H (2022). Risk factors and clinical outcomes of neonatal sepsis in manado tertiary referral hospital: a single-center study. Open Access Macedonian Journal of Medical Sciences.

[ref-34] Wilson E, Zeitlin J, Piedvache A, Misselwitz B, Christensson K, Maier RF, Norman M, Edstedt Bonamy A-K, EPICE Research Group (2018). Cohort study from 11 European countries highlighted differences in the use and efficacy of hypothermia prevention strategies after very preterm birth. Acta Paediatrica.

[ref-35] Yu Y-H, Wang L, Huang L, Wang L-L, Huang X-Y, Fan X-F, Ding Y-J, Zhang C-Y, Liu Q, Sun A-R, Zhao Y-H, Yao G, Li C, Liu X-X, Wu J-C, Yang Z-Y, Chen T, Ren X-Y, Li J, Bi M-R, Peng F-D, Geng M, Qiu B-P, Zhao R-M, Niu S-P, Zhu R-X, Chen Y, Gao Y-L, Deng L-P (2020). Association between admission hypothermia and outcomes in very low birth weight infants in China: a multicentre prospective study. BMC Pediatrics.

[ref-36] Zayeri F, Kazemnejad A, Ganjali M, Babaei G, Nayeri F (2007). Incidence and risk factors of neonatal hypothermia at referral hospitals in Tehran, Islamic Republic of Iran. Eastern Mediterranean Health Journal.

[ref-37] Zhou W, Lin ZL, Jia YS, Lin J (2010). Analysis of related factors of hypothermia in very low and extremely low birth weight infants. Chinese Journal of Perinatal Medicine.

