# Supplementary material for: Factors influencing hypothermia in very low/extremely low birth weight infants: a meta-analysis
Source: PeerJ. 2023 Feb 20;11:e14907. doi: 10.7717/peerj.14907 (PMC9948743; doi:10.7717/peerj.14907)
Supplement: Supplemental Information 1 — The appendix contains search strategies for 4 databases: PubMed, Embase, Cochrane Library and Web of Science. [file peerj-11-14907-s001.docx]

**1. PubMed**

Search **("infant, very low birth weight"[MeSH Terms] OR ("very low birth weight infant"[Title/Abstract] OR "infant very low birth weight"[Title/Abstract] OR "infants very low birth weight"[Title/Abstract] OR "very low birth weight infant"[Title/Abstract] OR "very low birth weight infants"[Title/Abstract] OR "Very-Low-Birth-Weight"[Title/Abstract]) OR ("infant, extremely low birth weight"[MeSH Terms] OR "extremely low birth weight infant"[Title/Abstract])) AND ("Hypothermia"[MeSH Terms] OR ("Hypothermia"[Title/Abstract] OR "hypothermia accidental"[Title/Abstract] OR "accidental hypothermia"[Title/Abstract] OR "accidental hypothermia"[Title/Abstract] OR "hypothermia accidental"[Title/Abstract])) AND (("relative"[Title/Abstract] AND "risk*"[Title/Abstract]) OR "relative risk"[Text Word] OR "risks"[Text Word] OR "cohort studies"[MeSH Terms:noexp] OR ("cohort"[Title/Abstract] AND "stud*"[Title/Abstract]))**

**2. Embase**

#1. 'very low birth weight'/exp

#2. 'infant, very low birth weight':ab,ti OR 'infant very low birth weight':ab,ti OR 'infants very low birth weight':ab,ti OR 'very low birth weight infant':ab,ti OR 'very low birth weight infants':ab,ti OR 'very-low-birth-weight':ab,ti

#3. 'extremely low birth weight'/exp

#4. 'extremely low birth weight infant':ab,ti OR 'infant, extremely low birth weight':ab,ti

#5. #1 OR #2 OR #3 OR #4

#6. 'hypothermia'/exp

#7. hypothermias':ab,ti OR 'hypothermia, accidental':ab,ti OR 'accidental hypothermia':ab,ti OR 'accidental hypothermias':ab,ti OR 'hypothermias, accidental':ab,ti

#8. #6 OR #7

#9. 'relative risk':ab,ti OR 'cohort studies':ab,ti

#10. #5 AND #8 AND #9

**3. Cochrane Library**

#1 MeSH descriptor: [Infant, Very Low Birth Weight] explode all trees

#2 (very low birth weight infant):ab,ti,kw OR (infant very low birth weight):ab,ti,kw OR (infants very low birth weight):ab,ti,kw OR (very low birth weight infant):ab,ti,kw OR (very low birth weight infants):ab,ti,kw OR (Very-Low-Birth-Weight):ab,ti,kw OR (extremely low birth weight infant):ab,ti,kw

#3 MeSH descriptor: [Infant, Extremely Low Birth Weight] explode all trees

#4 (extremely low birth weight infant):ab,ti,kw

#5 #1 or #2 or #3 or #4

#6 MeSH descriptor: [Hypothermia] explode all trees

#7 (Hypothermias):ab,ti,kw OR (Hypothermia, Accidental):ab,ti,kw OR (Accidental Hypothermia):ab,ti,kw OR (Accidental Hypothermias):ab,ti,kw OR (Hypothermias, Accidental):ab,ti,kw

#8 #6 or #7

#9 (relative risk):ab,ti,kw OR (cohort studies):ab,ti,kw

#10 #5 and #8 and #9

**4. Web of Science**

1: TS=(infant, very low birth weight OR very low birth weight infant OR infant very low birth weight OR infants very low birth weight OR very low birth weight infant OR very low birth weight infants OR Very-Low-Birth-Weight OR extremely low birth weight infant OR infant, extremely low birth weight OR extremely low birth weight infant)

2: TS=(Hypothermia OR Hypothermias OR Hypothermia, Accidental OR Accidental Hypothermia OR Accidental Hypothermias OR Hypothermias, Accidental)

3: TS=(relative risk OR cohort studies)

4: #1 AND #2 AND #3
