# Supplementary material for: Factors influencing hypothermia in very low/extremely low birth weight infants: a meta-analysis
Source: PeerJ. 2023 Feb 20;11:e14907. doi: 10.7717/peerj.14907 (PMC9948743; doi:10.7717/peerj.14907)
Supplement: Supplemental Information 2 — The appendix contains the results of the quality evaluation and risk bias assessment of the included literature. [file peerj-11-14907-s002.docx]

Newcastle-Ottawa Quality Assessment Scale (case control studies)

| Study | Year | Selection | | | | Comparability | Exposure | | |
| --- | --- | --- | --- | --- | --- | --- | --- | --- | --- |
|  |  | ① | ② | ③ | ④ | ⑤ | ⑥ | ⑦ | ⑧ |
| Gao et al | 2022 | 1 | 1 | 0 | 1 | 1 | 1 | 1 | 0 |
| Li et al | 2013 | 1 | 1 | 0 | 1 | 1 | 1 | 1 | 0 |
| Zhou et al | 2010 | 1 | 1 | 0 | 1 | 1 | 1 | 1 | 0 |
| Dong et al | 2021 | 1 | 1 | 0 | 1 | 1 | 1 | 1 | 0 |

①Is the case definition adequate?

②Representativeness of the cases

③Selection of Controls

④Definition of Controls

⑤Comparability of cases and controls on the basis of the design or analysis

⑥Comparability of cases and controls on the basis of the design or analysis

⑦Same method of ascertainment for cases and controls

⑧Non-Response rate

Newcastle-Ottawa Quality Assessment Scale (cohort studies)

| Study | Year | Selection | | | | Comparability | Outcome | | |
| --- | --- | --- | --- | --- | --- | --- | --- | --- | --- |
|  |  | ① | ② | ③ | ④ | ⑤ | ⑥ | ⑦ | ⑧ |
| Yong et al | 2020 | 1 | 1 | 1 | 1 | 2 | 1 | 1 | 1 |
| Maria et al | 2014 | 1 | 1 | 1 | 1 | 2 | 1 | 1 | 1 |
| Akter et al | 2013 | 1 | 1 | 1 | 1 | 2 | 1 | 1 | 1 |
| Miller et al | 2011 | 1 | 1 | 1 | 1 | 2 | 1 | 1 | 1 |
| Emilija et al | 2018 | 1 | 1 | 1 | 1 | 2 | 1 | 1 | 1 |
| Na et al | 2019 | 1 | 1 | 1 | 1 | 2 | 1 | 1 | 1 |

①Representativeness of the exposed cohort

②Representativeness of the exposed cohort

③Ascertainment of exposure

④Demonstration that outcome of interest was not present at start of study

⑤Comparability of cohorts on the basis of the design or analysis

⑥Assessment of outcome

⑦Assessment of outcome

⑧Assessment of outcome
