# Supplementary material for: Factors influencing hypothermia in very low/extremely low birth weight infants: a meta-analysis
Source: PeerJ. 2023 Feb 20;11:e14907. doi: 10.7717/peerj.14907 (PMC9948743; doi:10.7717/peerj.14907)
Supplement: Supplemental Information 5 [file peerj-11-14907-s005.docx]

**What is already known**

- As one of the vital signs, body temperature is important to reflect the physical condition and prognosis. Due to the imperfect development of subcutaneous fat and thermoregulatory center in very low/extremely low birth weight infants, the incidence of hypothermia is high, which poses a serious test to the life safety of very low/extremely low birth weight infants.
- Current research related to hypothermia in very low/extremely low birth weight infants have focused on quality of care improvement and influencing factors, and there is a lack of scientific integration of related findings.

**What this paper adds**

- Pubmed and other databases were applied to search for case-control or cohort studies on factors influencing the occurrence of hypothermia in very low/extremely low birth weight infants.
- Scientific and systematic integration of factors influencing the occurrence of hypothermia in very low/extremely low birth weight infants.
